# Relationship of Soluble Klotho and Early Stage of Diabetic Nephropathy: A Systematic Review and Meta-Analysis

**DOI:** 10.3389/fendo.2022.902765

**Published:** 2022-05-27

**Authors:** Caihong Xin, Xin Sun, Zheng Li, Tianshu Gao

**Affiliations:** ^1^ Liaoning University of Traditional Chinese Medicine, Shenyang, China; ^2^ Department of Endocrinology and Metabolism, Fourth People’s Hospital of Shenyang, Shenyang, China; ^3^ Department of Endocrinology and Metabolism, First Affiliated Hospital of Soochow University, Suzhou, China

**Keywords:** Klotho, diabetic nephropathy, DN, meta- analyses, Systematic (Literature) Review

## Abstract

**Background:**

Diabetic nephropathy (DN) is a chronic microvascular complication caused by long-term hyperglycemia in patients with diabetes and an important cause of end-stage renal disease. Although some studies have shown that soluble Klotho(sKlotho) levels of patients with DN are lower than those without DN, in the early stage of patients with DN with normal renal function and albuminuria, the change in sKlotho is still controversial.

**Aim:**

This meta-analysis was conducted to statistically evaluate sKlotho levels in patients with DN.

**Methods:**

We searched the following electronic databases: Web of Science, Embase, PubMed, Google Scholar, and China National Knowledge Infrastructure (CNKI). The following search terms were used for the title or abstract: “diabetic kidney disease”, “diabetic nephropathy”, OR “DN” in combination with “Klotho”. The meta-analysis results were presented as standardized mean differences (SMDs) with corresponding 95% confidence intervals (CIs).

**Results:**

Fourteen articles were included in the meta-analysis. In our meta-analysis, we found that the sKlotho level in patients with DN was significantly lower than that in patients without DN (SMD: -1.52, 95% CI [-2.24, -0.80]), and it was also significantly lower in the early stage of DN (SMD: -1.65, 95% CI [-2.60, -0.70]).

**Conclusions:**

This systematic review was the first to evaluate the relationship between sKlotho levels and DN. The sKlotho level was significantly lower in the early stages of DN, indicating that sKlotho might be a new biomarker of DN in the future.

## Introduction

Diabetic nephropathy (DN) is a chronic microvascular complication caused by long-term hyperglycemia in patients with diabetes and an important cause of chronic kidney disease (CKD). The pathogenesis of DN is complex. It is associated with hyperglycemia and insulin resistance in patients with diabetes. It has also been associated with abnormal lipid metabolism, inflammation, and oxidative stress (1, 2). The onset of DN is insidious, and there are often no obvious clinical manifestations in its early stages. Once it enters the clinical stage of nephropathy, renal lesions will be irreversible, which will delay the treatment of the disease. Therefore, early diagnosis and treatment are effective for treating DN (3). Microalbuminuria is considered to be the most common early manifestation of DN. However, microalbuminuria is often intermittent and fluctuating, and the early stage of nephropathy is often not accompanied by obvious symptoms, which can easily be ignored and missed (4). Therefore, new biomarkers are urgently required to assist in the early diagnosis of DN.

Klotho is a protein with anti-aging activity discovered by Japanese scientists in spontaneously hypertensive rats in 1997 (5). The Klotho gene is about 50 kb long, and two mRNA transcripts can arise through alternative splicing: one generates the type I transmembrane protein (130 kDa), the other is assumed to generate a secreted protein (70 kDa). Transmembrane Klotho protein is expressed mainly in choroid plexus epithelial cells of the brain and the distal convoluted tubules of the kidney. The extracellular region of transmembrane Klotho protein can be cleaved by α- and β-secretases, and eventually finds its way into blood, urine and cerebrospinal fluid. This cleaved Klotho protein is commonly known as the soluble Klotho(sKlotho) (6, 7). Previous studies have mainly focused on anti-aging functions, such as cell survival, proliferation, and apoptosis (8). In recent years, it has also been found to be involved in the regulation of energy metabolism. As the concentration of sKlotho increased, it exerted a protective effect on renal endothelial cells (9, 10). However, in the early stage of DN in patients with normal renal function, the change in sKlotho remains controversial (11–15). Therefore, this study aimed to investigate the relationship between the sKlotho levels and DN, especially its relationship with the early stage of DN to guide clinical treatment and prognosis.

## Methods

### Search

We searched the following electronic databases: Web of Science, Embase, PubMed, Google Scholar, and China National Knowledge Infrastructure (CNKI). The following search terms were used for the title or abstract: “diabetic kidney disease” OR “diabetic nephropathy” in combination with the term “Klotho.” The retrieval time was limited to 1980–2020, and the language was limited to English and Chinese. We also checked the references of the retrieved articles to avoid missing additional eligible studies. We did not search for any unpublished studies. The registration number for the systematic review and meta-analysis was CRD 42022309103 in PROSPERO. A complete list of the preferred reporting items for systematic reviews and meta-analyses is provided in the **Supplementary Data (S1)**.

### Inclusion Criteria

The studies included in this meta-analysis met the following criteria: (1) detailed data about the sKlotho levels in patients with diabetes and DN; (2) patients with different stages of DN; and (3) multiple studies of the same author and department; only the study with the largest sample size was selected.

The degree of DN was based on the urine albumin-creatinine ratio (UACR) and chronic kidney disease-Kidney Disease Outcomes Quality Initiative (CKD-KDOQI) criteria. Patients with diabetes were categorized into three groups according to the UACR: UACR < 30 mg/g creatinine (normoalbuminuria), UACR 30–299 mg/g creatinine (microalbuminuria), and UACR ≥ 300 mg/g creatinine (macroalbuminuria). The early stage of DN was defined as microalbuminuria or CKD stage 1–2.

### Data Extraction and Risk of Bias

Two reviewers independently searched according to the search strategy and independently read the title and abstract according to the search results for preliminary screening to exclude the study that did not meet the inclusion criteria. The full text of the papers were analyzed to determine whether they meet the inclusion criteria. Two reviewers can contact and crosscheck the author if the information is incomplete. If the conclusions of the two evaluators were not consistent, the differences were resolved through discussion. If the discussion failed to resolve the differences, it was judged and arbitrated by a third researcher. The Newcastle-Ottawa Scale (NOS) is a risk of a bias assessment tool for observational studies recommended by the Cochrane Collaboration (16, 17). The quality of the included studies was evaluated according to the NOS. The NOS includes three aspects: the selection method of the case and control groups, comparability of the case and control groups, and evaluation method of exposure. The NOS ranged from zero to nine stars, and quality was based on star scores.

### Statistical Analysis

The data were expressed as standardized mean differences (SMD) and 95% confidence intervals (CIs). Heterogeneity among the included studies was assessed using the I^2^ statistic. If I^2^ was < 50%, the heterogeneity among studies was low or moderate, and the fixed-effect model was adopted. Otherwise, if I^2^ was > 50%, the random-effects model was used for analysis. Sensitivity analysis was performed to judge the stability of the results. The Begg’s and Egger’s tests were used to identify publication bias. Statistical significance was set at *P* < 0.05. Data were analyzed using Stata version 12.0 (College Station, TX, USA).

## Results

**Figure 1:**
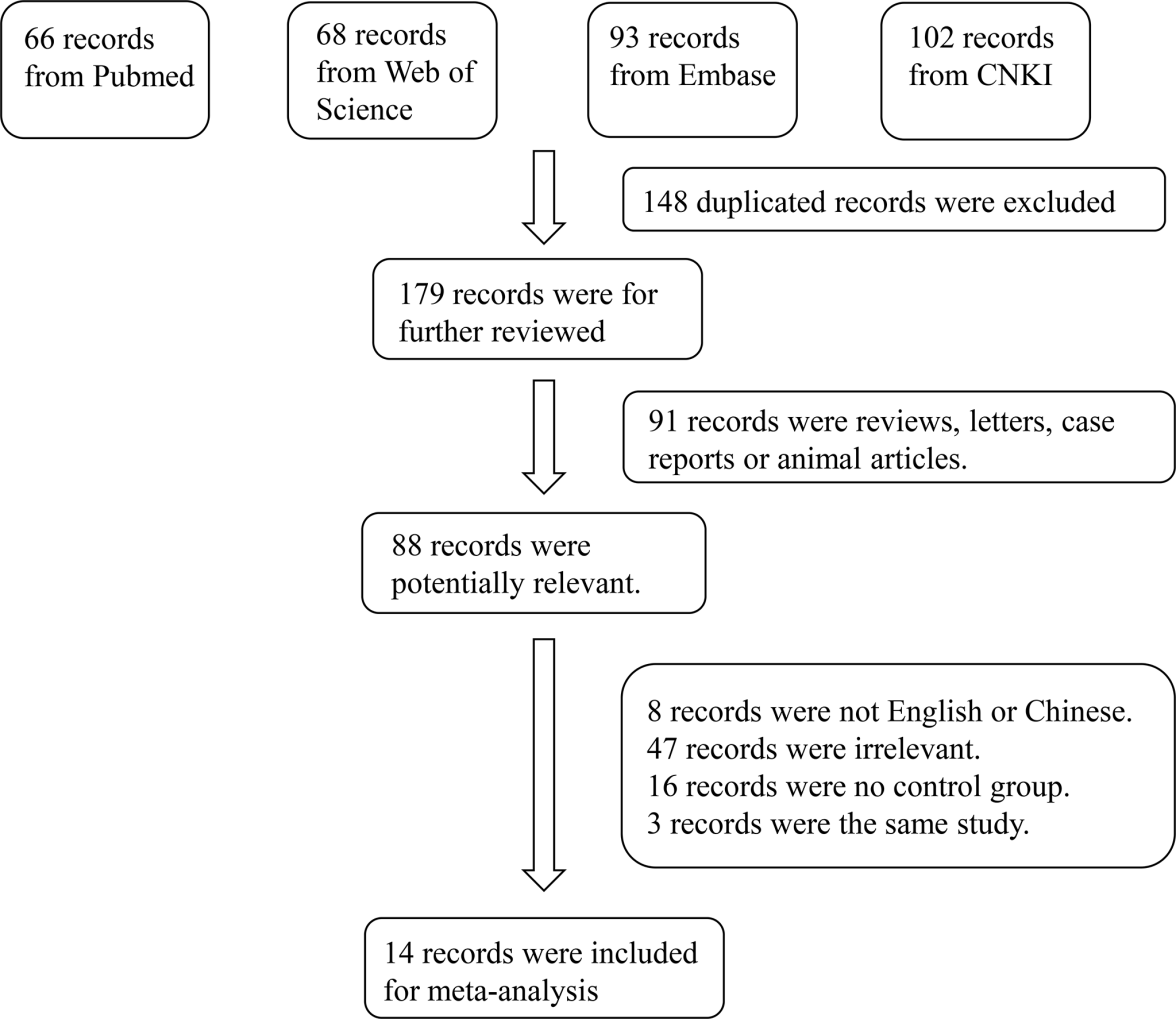
Flowchart of the detailed procedure for the inclusion or exclusion of selected studies.

A total of 329 studies were retrieved from the databases. After screening, 14 articles were selected (11–14, 18–27). The flow diagram of the article selection process is shown in **Figure 1**. The characteristics of the selected studies are summarized in **Table 1**.

**Table 1:** Study characteristics of the published studies included in the meta-analysis.

Author	Publication Year	Study Period	Region	Study Design	Study Sample	Sex	Age	Details	NOS
Kacso IM (11)	2012	–	Romania	Case-control study	146 patients with type 2 diabetes and 43 healthy controls	patients with type 2 diabetes: 87 males; healthy controls: 20 males.	patients with type 2 diabetes: mean age 63.38 ± 12.20 years; healthy controls: mean age 61.07 ± 11.47 years.	The diabetic patients were categorized into 3 groups according to UACR. The mean duration of diabetes was 8.8 ± 5.6 years. Ninety-two of the diabetic patients were on insulin treatment, 43 were treated with metformin, and 44 with sulfonylureas.	5
Lee EY (18)	2014	February 2010 - February 2012	Korea	Case-control study	147 patients with type 2 diabetes and 43 healthy controls	patients with type 2 diabetes: 63 males; healthy controls: 14 males.	patients with type 2 diabetes: mean age 56.6 ± 10.6 years; healthy controls: mean age 50.9 ± 7.6 years.	The diabetic patients were categorized into 3 groups according to UACR. The mean duration of diabetes was 7.8 ± 5.9 years.	6
Wu C (12)	2014	April 2010 - July 2013	China	Case-control study	462 patients with type 2 diabetes and 160 healthy controls	patients with type 2 diabetes: 214 males; healthy controls: 78 males.	patients with type 2 diabetes: mean age 52.9 ± 6.3 years; healthy controls: mean age 51.8 ± 6.5 years.	The diabetic patients were categorized into 3 groups according to UACR. The mean duration of diabetes was 7.7 ± 4.2 years.	7
Dogan B (13)	2016	February 2012 - March 2013	Turkey	Case-control study	147 patients with type 1 diabetes and 76 healthy controls	patients with type 1 diabetes: 65 males; healthy controls: 33 males.	patients with type 1 diabetes: mean age 34.1 ± 9.2 years; healthy controls: mean age 33.9 ± 9.1 years.	–	7
Inci A (14)	2016	January 2014 - June 2014	Turkey	Case-control study	109 patients with type 2 diabetes and 32 healthy controls	patients with type 2 diabetes: 62 males; healthy controls: 12 males.	patients with type 2 diabetes: mean age 61.63 ± 9.77 years; healthy controls: mean age 49.53 ± 7.32 years.	The diabetic patients were categorized into 3 groups according to UACR.	7
Maltese G (19)	2017	–	London	Observational study	78 patients with type 1 diabetes	patients with type 1 diabetes: 43 males	patients with microalbuminuria: mean age 54.4 ± 11.6 years; controls: mean age 43.3 ± 9.6 years.	The diabetic patients were categorized into 2 groups according to UACR. The mean duration of diabetes was 30.9 ± 10.0 years	5
Nie F (20)	2017	2014 - 2016	China	Observational study	96 patients with type 2 diabetes, 90 patients with diabetic nephropathy and 106 healthy controls	patients with type 2 diabetes: 42 males; patients with diabetic nephropathy: 43 males; healthy controls: 52 males	patients with type 2 diabetes: 59.5(53-71) years; patients with diabetic nephropathy: 62(57.3-73.5) years; healthy controls: 57(47-63) years.	The diabetic patients were categorized into 3 groups according to UACR.	6
Ye M (21)	2017	June 2016 - January 2017	China	Case-control study	160 patients with type 2 diabetes and 80 healthy controls	patients with type 2 diabetes: 101 males; healthy controls: 47 males.	patients with type 2 diabetes: mean age 52.85 ± 11.15 years; healthy controls: mean age 52.47 ± 12.15 years.	All the patients were newly diagnosed and categorized into 2 groups according to UACR.	6
Fountoulakis N (22)	2018	2004-2006	London	Observational study	101 patients with type 2 diabetes	patients with type 2 diabetes: 60 males	mean age (range) was 60 (40-82) years.	The mean duration of diabetes was 9.8 ± 6.6 years, with a mean eGFR of 90.4 ± 20.0 ml/min. The diabetic patients were categorized into 2 groups according to UACR.	8
Zhang L (23)	2018	January 2016 - January 2018	China	Case-control study	127 patients with type 2 diabetes, 106 patients with diabetic nephropathy and 140 healthy controls	patients with type 2 diabetes: 59 males; patients with diabetic nephropathy: 55 males; healthy controls: 77 males	patients with type 2 diabetes: 52.5 (41–71) years; patients with diabetic nephropathy: 54.6 (42–77) years; healthy controls: 52.9 (39–71) years.	–	7
Li Q (24)	2019	February 2017 - September 2017	China	Case-control study	68 patients with type 2 diabetes and 22 healthy controls	patients with type 2 diabetes: 39 males; healthy controls: 12 males.	patients with type 2 diabetes: mean age 48.1 ± 6.3 years; healthy controls: mean age 45.5 ± 4.2 years.	The diabetic patients were categorized into 3 groups according to UACR.	6
Chen J (25)	2020	February 2015 - August 2018	China	Case-control study	160 patients with type 2 diabetes and 60 healthy controls	patients with type 2 diabetes: 70 males; healthy controls: 20 males.	patients with type 2 diabetes: mean age 57.8 ± 5.6 years; healthy controls mean age 50.2 ± 6.9 years.	The diabetic patients were categorized into 3 groups according to UACR.	7
Tian Q (26)	2020	November 2016 - November 2018	China	Case-control study	174 patients with type 2 diabetes and 55 healthy controls	patients with type 2 diabetes: 90 males; healthy controls: 26 males.	patients with type 2 diabetes: mean age 58.0 ± 7.9 years; healthy controls: mean age 56.1 ± 7.4 years.	The mean duration of diabetes was 10.9 ± 3.7 years. The diabetic patients were categorized into 3 groups according to UACR.	6
Nie L (27)	2021	February 2017 - May 2018	China	Case-control study	80 patients with type 2 diabetes and 50 healthy controls	patients with type 2 diabetes: 36 males; healthy controls: 23 males.	patients with type 2 diabetes: mean age 58.6 ± 5.58 years; healthy controls: mean age 58.27 ± 5.35 years.	The mean duration of diabetes was 6.3 ± 0.7 years. Diabetic nephropathy was diagnosed by biopsy.	5

NOS, Newcastle-Ottawa Scale; UACR, urine albumin-creatinine ratio.

### Results of the Meta-Analysis

**Figure 2:**
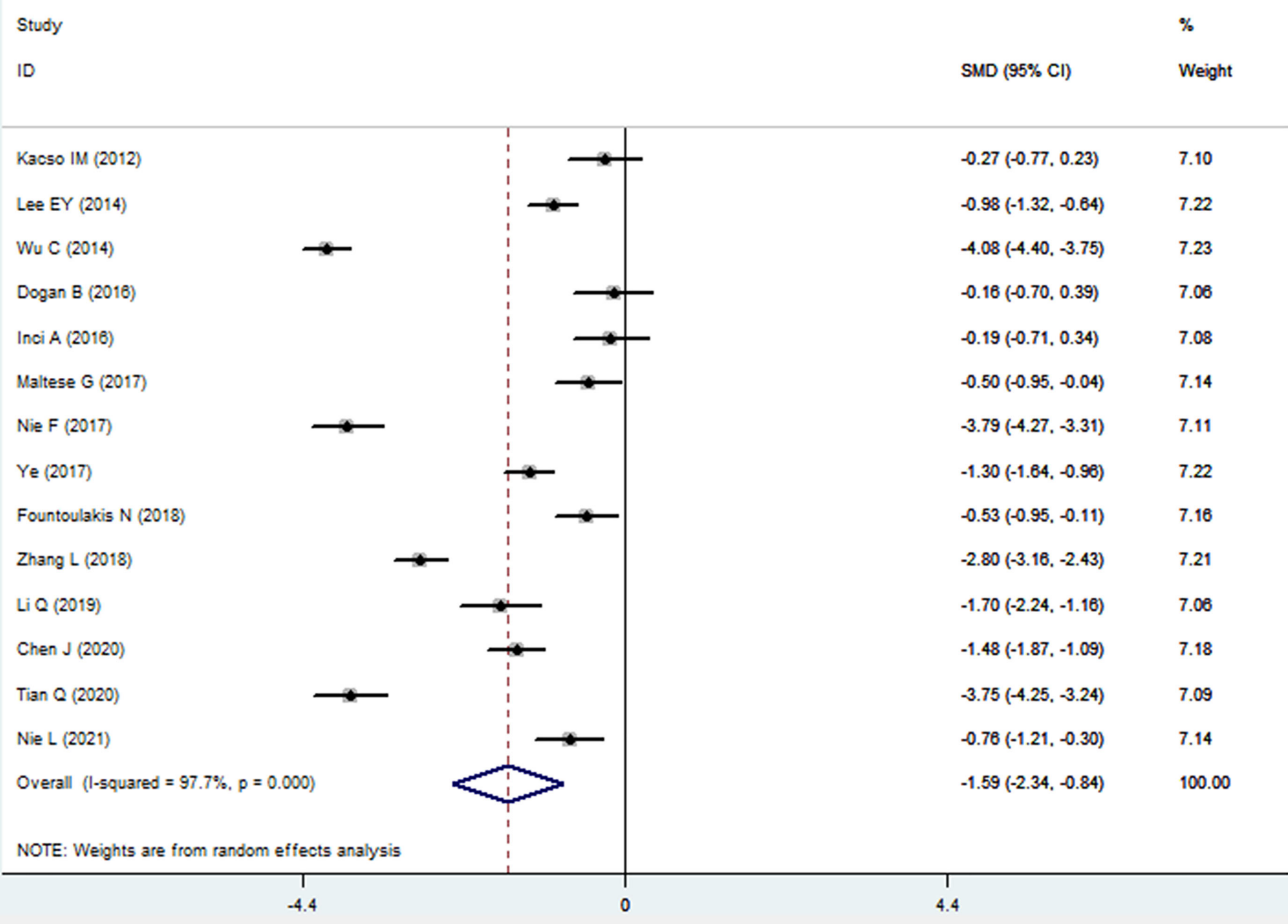
Forest plots and funnel plots of sKlotho level in patients with diabetic nephropathy compared to diabetes. Diamond represents the pooled SMDs at 95% CI. SMD, standardized mean difference; CI, confidence interval.

**Figure 3:**
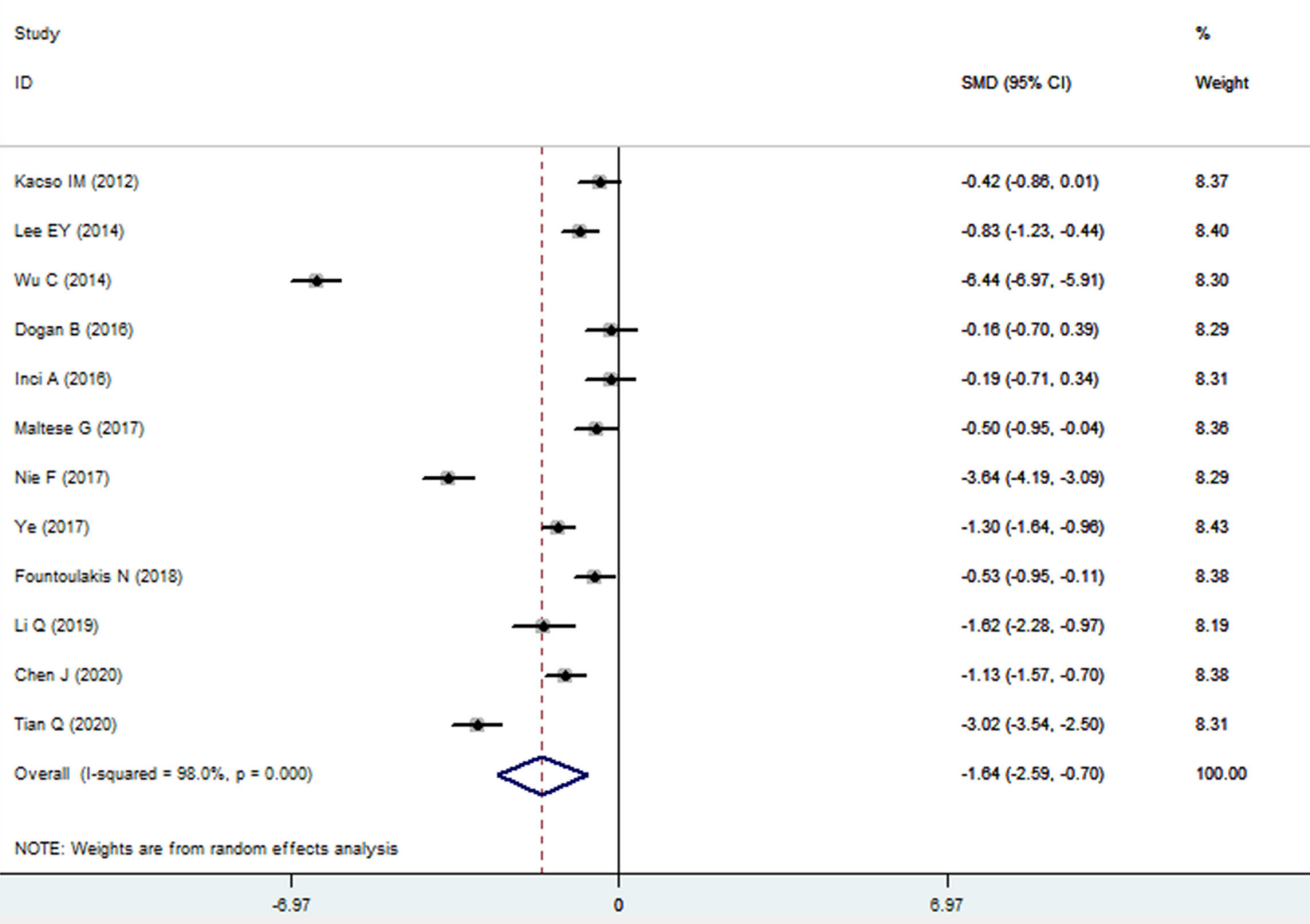
Forest plots and funnel plots of sKlotho level in patients with early stage of diabetic nephropathy compared to diabetes without diabetic nephropathy. Diamond represents the pooled SMDs at 95% CI. SMD, standardized mean difference; CI, confidence interval.

**Figure 4:**
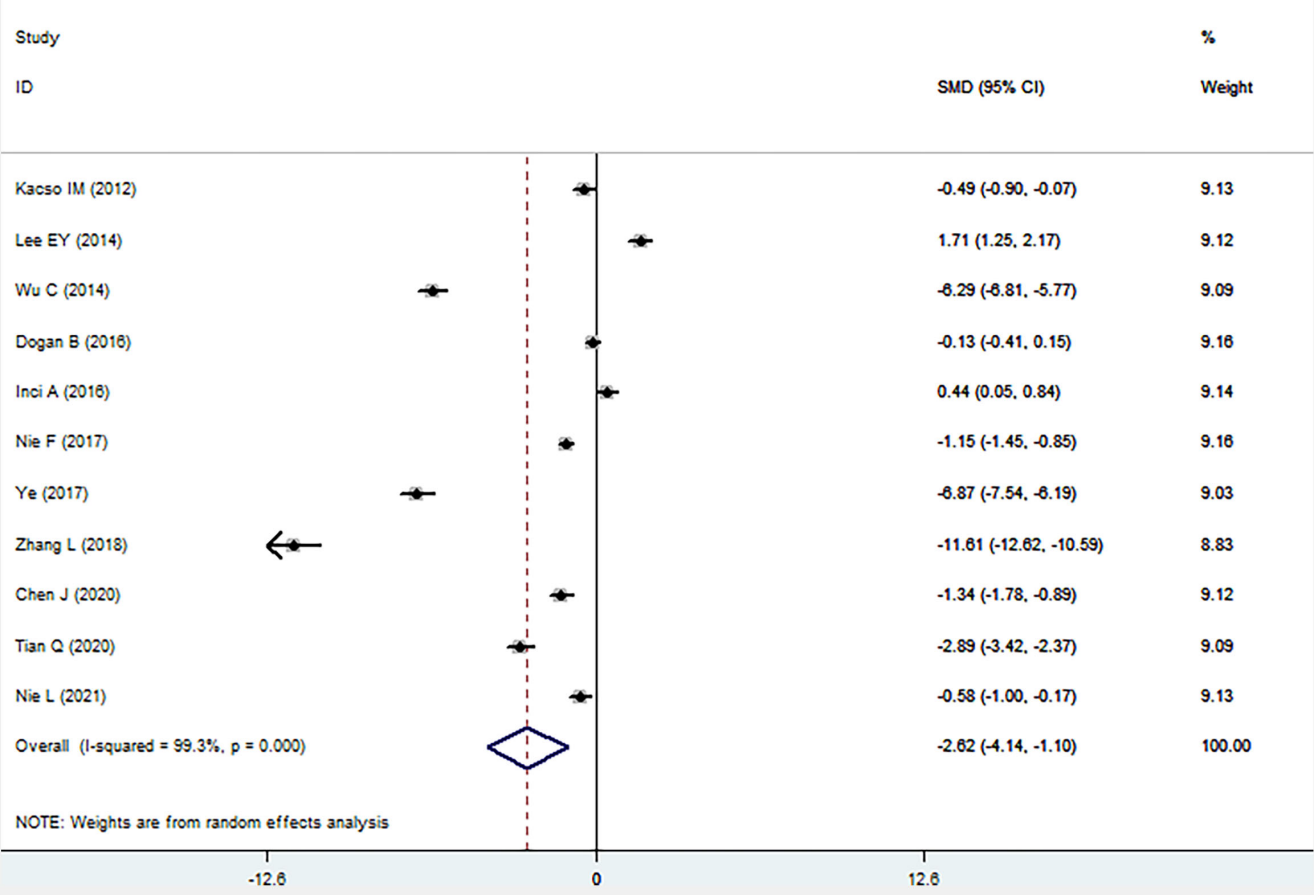
Forest plots and funnel plots of sKlotho level in patients with diabetes compared to the controls. Diamond represents the pooled SMDs at 95% CI. SMD, standardized mean difference; CI, confidence interval.

The sKlotho level in patients with DN was significantly lower than that in patients with diabetes (SMD: -1.52, 95% CI [-2.24, -0.80]). Forest plots of the sKlotho levels in patients with DN compared to those with diabetes are presented in **Figure 2**. Moreover, there was a significant difference in the sKlotho levels between patients with diabetes without DN and those with early stage of DN (SMD: -1.65, 95% CI [-2.60, -0.70]). Forest plots of the sKlotho levels are shown in **Figure 3**. Compared with the controls, the sKlotho level was also significantly lower in patients with diabetes (SMD: -2.12, 95% CI [-4.14, -1.10], **Figure 4**).

### Sensitivity Analysis and Publication Bias

Sensitivity analysis was performed to examine the influence of each study. We found no significant difference between the sensitivity analysis results and our previous estimates, indicating that the data of a single study had a little overall impact. Thus, it can be inferred that the results of this meta-analysis were stable. (**Figures S1**-**S3**). A careful and comprehensive search was performed for articles obtained from the database. In addition, the Begg’s and Egger’s tests were performed to determine whether there was a potential publication bias in the reviewed study. The results (*P* > 0.05) indicated no publication bias.

## Discussion

This systematic review was the first to evaluate the relationship between the sKlotho levels and DN in patients with diabetes. Although some studies have shown that the sKlotho levels of patients with DN are lower than those without DN, in the early stage of patients with DN with normal renal function and albuminuria, the change in sKlotho is still controversial. Fourteen independent studies were included in the meta-analysis. We concluded that the sKlotho level in patients with DN was significantly lower than that in patients without DN (SMD: -1.52, 95% CI [-2.24, -0.80]), and it was also significantly lower in the early stages of DN (SMD: -1.65, 95% CI [-2.60, -0.70]).

Diabetic nephropathy is characterized by changes in the structure and function of the glomeruli. The disease is reversible at an early stage and irreversible in patients with persistent proteinuria. The International Diabetes Federation predicts that by 2030, the total number of patients with type 2 diabetes will reach 439 million worldwide (28). The incidence of diabetes-related complications is also increasing with an increase in the number of patients with diabetes. Identifying serum biomarkers for the early diagnosis of DN is of great significance for improving the prognosis of patients with diabetes. Determination of renal function is an important means of diagnosing DN (29). However, there are still some changes in renal function indices in some patients. In recent years, the role of Klotho in diabetes and kidney disease has attracted increasing attention (30).

**Figure 5:**
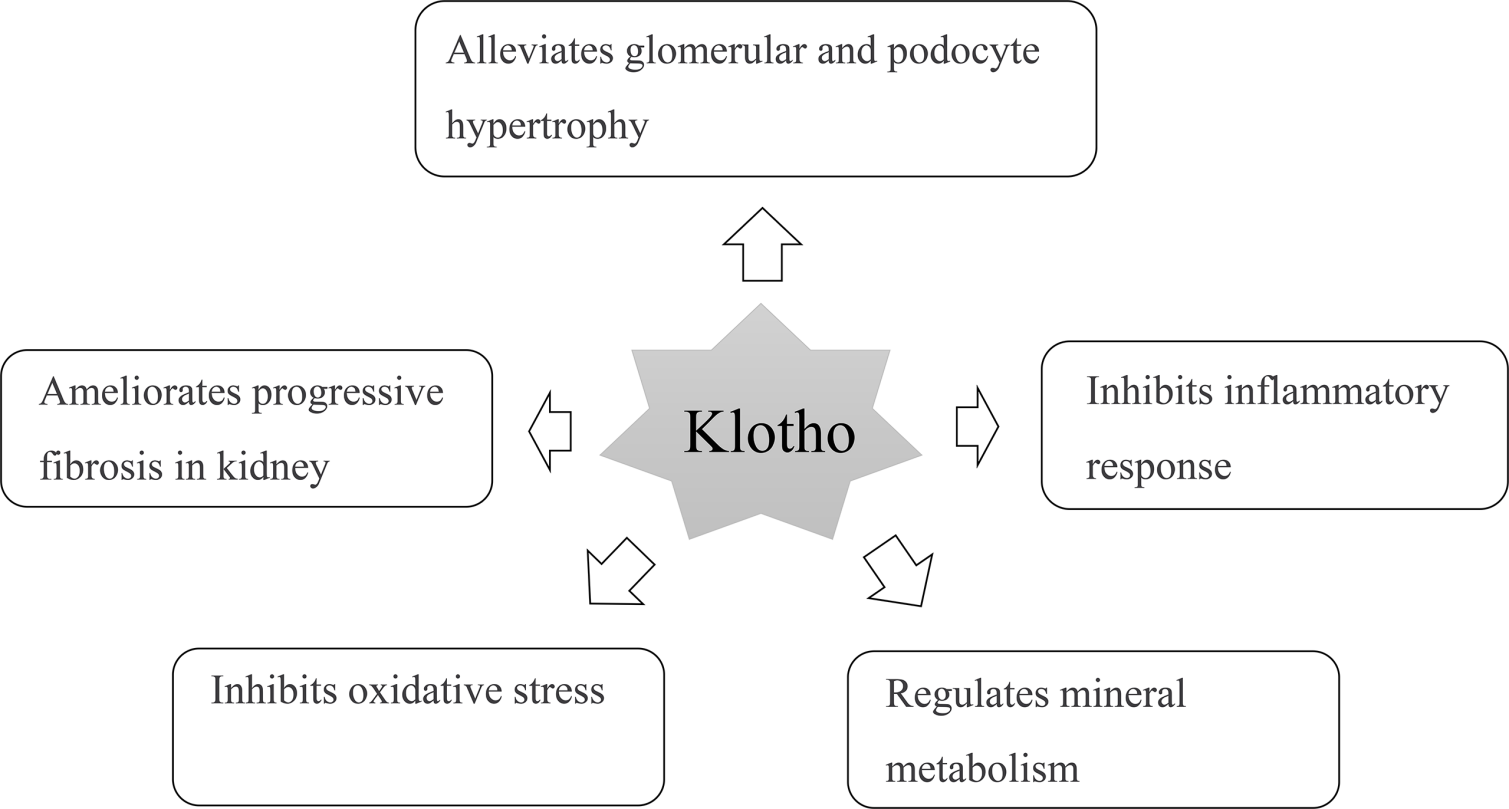
Multifactorial effects of Klotho in the pathology of diabetic nephropathy.

Klotho protein is a transmembrane protein and mainly expressed in the kidney, heart, and brain and can inhibit the inflammatory response (31). It protects islet β-cell function and can promote insulin secretion, thereby reducing blood glucose and delaying the process of renal disease (**Figure 5**) (32). Supplementing exogenous α-Klotho can reduce hyperglycemia injury by promoting glycogen storage, inhibiting gluconeogenesis, improving insulin sensitivity in type 2 diabetes, anti-inflammatory, antioxidant stress, and inhibiting fibrosis; thus, protecting the kidney (33–35). Klotho attenuates diabetic nephropathy in db/db mice and ameliorates high glucose-induced injury of human renal glomerular endothelial cells (36). In addition, Klotho can inhibit the renin-angiotensin-aldosterone system, and the nuclear factor kappa B (NF-κB) signaling pathway inhibits renal fibrosis caused by the inflammatory response. Therefore, the consumption of Klotho in patients with DN is relatively high, resulting in a decrease in the Klotho levels (37, 38). Animal experiments have shown that mice lacking the Klotho gene show significant changes, such as endothelial cell injury and abnormal energy metabolism. After sKlotho supplementation, the expression of nitric oxide in vascular endothelial cells increases, reverses vascular inflammatory reactions, and protects renal function (39). Exogenous Klotho attenuates high glucose (HG)-induced profibrotic genes, TGF-β signaling and cell hypertrophy in rat renal interstitial fibroblasts (NRK-49F) cells. Moreover, Klotho attenuates HG-induced fibronectin expression and cell hypertrophy *via* the ERK1/2 and p38 kinase-dependent pathways (40). Klotho protein overexpression attenuates renal hypertrophy and glomerular injury in this mouse model of diabetic nephropathy. Klotho overexpression attenuated renal hypertrophy, albuminuria, glomerular mesangial expansion, and endothelial glycocalyx loss in the AKITA mice (41). In our meta-analysis, we found that even in the early stages of DN, the sKlotho level was significantly lower in patients with diabetes without DN. We hope that sKlotho is a more sensitive biomarker during the early stages of DN.

This meta-analysis aimed to statistically evaluate the sKlotho levels in patients with DN. However, this study had some limitations. In different studies, the duration of diabetes and severity of the disease were variant. Meanwhile, the overall quality of the studies was not high. All of these factors may have affected the results; therefore, the findings of this meta-analysis should be interpreted cautiously, as further research is needed.

## Conclusion

This systematic review was the first to evaluate the relationship between the sKlotho levels and DN. The sKlotho level was significantly lower in the early stages of DN, indicating that sKlotho might be a new biomarker of DN in the future.

## Data Availability Statement

The original contributions presented in the study are included in the article/**Supplementary Material**. Further inquiries can be directed to the corresponding author.

## Author Contributions

TG designed the study. CX searched databases and collected the data. XS and ZL assessed the quality of the study. XS performed the analysis. TG and CX wrote the manuscript. All authors contributed to this systematic review and meta-analysis. All authors contributed to the article and approved the submitted version.

## Conflict of Interest

The authors declare that the research was conducted in the absence of any commercial or financial relationships that could be construed as a potential conflict of interest.

## Publisher’s Note

All claims expressed in this article are solely those of the authors and do not necessarily represent those of their affiliated organizations, or those of the publisher, the editors and the reviewers. Any product that may be evaluated in this article, or claim that may be made by its manufacturer, is not guaranteed or endorsed by the publisher.

## Supplementary Material

Supplementary Table 1 Preferred reporting items for systematic review and meta-analyses (PRISMA) checklist.

Supplementary Figure 1 The sensitivity analysis results of sKlotho level in patients with diabetic nephropathy compared to diabetes.

Supplementary Figure 2 The sensitivity analysis results of sKlotho level in patients with early stage of diabetic nephropathy compared to diabetes without diabetic nephropathy.

Supplementary Figure 3 The sensitivity analysis results of sKlotho level in patients with diabetes compared to the controls.
