# Supplementary figures and images for: Relationship of Soluble Klotho and Early Stage of Diabetic Nephropathy: A Systematic Review and Meta-Analysis
Source: Front Endocrinol (Lausanne). 2022 May 27;13:902765. doi: 10.3389/fendo.2022.902765 (PMC9186104; doi:10.3389/fendo.2022.902765)

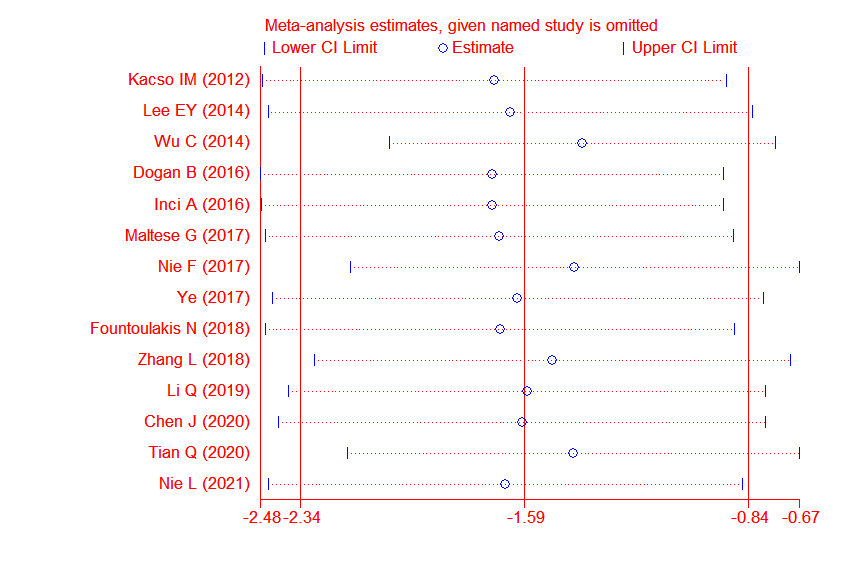

Supplement: Supplementary Figure 1 — The sensitivity analysis results of sKlotho level in patients with diabetic nephropathy compared to diabetes. [file Image_1.tif]

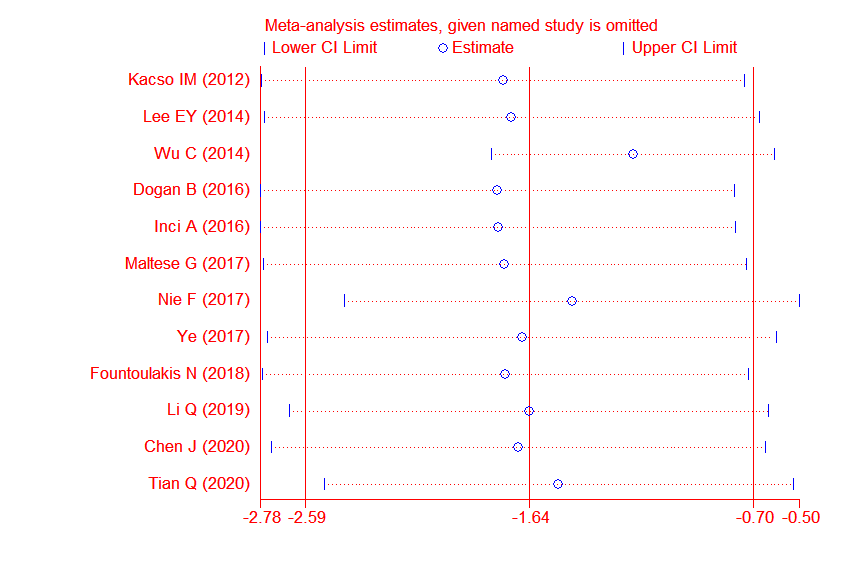

Supplement: Supplementary Figure 2 — The sensitivity analysis results of sKlotho level in patients with early stage of diabetic nephropathy compared to diabetes without diabetic nephropathy. [file Image_2.tif]

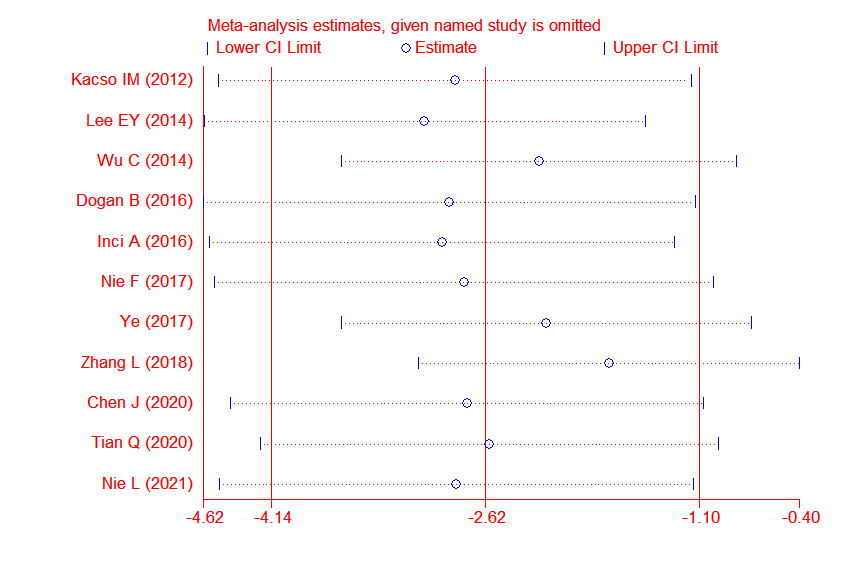

Supplement: Supplementary Figure 3 — The sensitivity analysis results of sKlotho level in patients with diabetes compared to the controls. [file Image_3.tif]
